# Plasticity of fertilization rates under varying temperature in the broadcast spawning mussel, *Mytilus galloprovincialis*

**DOI:** 10.1002/ece3.2375

**Published:** 2016-08-23

**Authors:** Angela R. Eads, Jonathan P. Evans, Winn Jason Kennington

**Affiliations:** ^1^ Centre for Evolutionary BiologySchool of Animal BiologyUniversity of Western AustraliaCrawleyWestern AustraliaAustralia

**Keywords:** Broadcast spawn, climate change, fertilization, mussel, *Mytilus galloprovincialis*, ocean warming, phenotypic plasticity

## Abstract

Oceans are a huge sink for the increased heat associated with anthropogenic climate change, and it is vital to understand the heat tolerance of marine organisms at all life stages to accurately predict species’ responses. In broadcast spawning marine invertebrates, reproduction is a vulnerable process in which sperm and eggs are released directly into the open water. Gametes are then exposed to fluctuating environmental conditions that may impact their fertilizing capacity. Using the broadcast spawning Mediterranean mussel, *Mytilus galloprovincialis,* as a model species, we performed blocks of factorial mating crosses to assess the variance in fertilization rates among individuals under both ambient and elevated temperatures. Overall, we found a small, but significant decline in fertilization rates with elevated temperatures. However, there was substantial plasticity in responses, with particular mussels having increased fertilization under elevated temperatures, although the majority showed decreased fertilization rates. Our results suggest possible future reproductive costs to ocean warming in *M. galloprovincialis*, although it is also possible that genetic variation for thermal sensitivity may allow for adaptation to changing environmental conditions.

## Introduction

Global warming could be more accurately described as ocean warming, as the oceans have stored nearly all of the excess heat associated with anthropogenic greenhouse gas emissions (Levitus 2005; Rintoul 2011; Durack et al. 2014; Bureau of Meteorology 2015). As average ocean temperatures continue to increase, and there are more extreme temperature peaks (Pearce and Feng 2013), it is vital that we understand the capacity of marine organisms to tolerate elevated temperatures. Early reproductive stages of marine organisms are likely to be sensitive to environmental stressors, and any negative impacts at these early stages could lead to bottlenecks for species’ survival (Byrne 2011). Sexual reproduction in marine broadcast spawners involves gametes being released directly into the water column, where sperm then have to locate and fertilize eggs in this exposed and turbulent three‐dimensional environment. The ability of marine gametes to locate each other by detecting and responding to chemical signals may be interrupted or impaired by fluctuating and changing ocean conditions, further impacting on their overall fertilization capacity. Any disruption to this fundamental reproductive process may have serious implications for offspring fitness, as well as species’ adaptability and persistence (Uthicke et al. 2009; Byrne 2012).

Studies investigating the effects of temperature on fertilization in marine invertebrates are highly variable both in methodology and result (Byrne 2012). Many studies of intertidal species have revealed some tolerance to temperature fluctuations (Mita et al. 1984; Clotteau and Dubé 1993; Bassim et al. 2002; Lewis et al. 2002; Lee et al. 2004; Byrne et al. 2010), but other studies have identified a significant decrease in fertilization rates when temperatures are elevated up to 6°C above ambient (Rupp 1973; O'Connor and Mulley 1977; Greenwood and Bennett 1981; Desrosiers et al. 1996; Sewell and Young 1999; Kupriyanova and Havenhand 2005; Negri et al. 2007; Byrne et al. 2009; Parker et al. 2010; Ericson et al. 2012). Temperature increases of this magnitude in marine habitats are realistic for the coming century, particularly considering the predicted increases in marine heat wave occurrence and length along with more extreme peaks in temperature fluctuations (IPCC 2013; Pearce and Feng 2013).

The cosmopolitan Mediterranean mussel, *Mytilus galloprovincialis* (L.)*,* is a sessile broadcast spawner, so their responses to environmental fluctuations are largely unmitigated by behavior. Their geographic and upper intertidal distributions are likely constrained by physiological limits such as thermal tolerance (Gardner 1992; Anestis et al. 2007; Jansen et al. 2009; Rayssac et al. 2010). Indeed, the range of *M. galloprovincialis* is limited to the southern half of the Australian coastline below 32° S latitude and throughout the intertidal zone (Suchanek 1978; Dias et al. 2014). Coastal habitats within this region, where large populations of both wild and farmed *M. galloprovincialis* are found, are affected by unprecedented rates of increase in ocean temperatures. The rate of ocean warming around Australia has been greatest off the southwest and southeast coasts, attributable to the poleward‐flowing currents (Pearce and Feng 2007; Lough et al. 2012). Consequently, this has generated ocean warming “hot spots” where warming is occurring 90% faster than in the rest of the oceans (Hobday and Pecl 2013). Furthermore, the ocean temperature increase off the coast of Western Australia has been, and will continue to be, most pronounced in winter, when the warm southward‐flowing Leeuwin Current is strongest (Caputi et al. 2009; Feng et al. 2012; Lough et al. 2012). This corresponds with *M. galloprovincialis’* spawning season in Australia (Wilson and Hodgkin 1967) and may have a direct impacts on fertilization rates, gamete‐level interactions (e.g., interfere with chemical cues used for sperm activation, chemotaxis, or egg receptivity; Zimmer and Riffell 2011; Evans and Sherman 2013), and ultimately survival.

Our study aimed to assess the impact of a realistic ocean temperature increase on the fertilizing capacity of *M. galloprovincialis* gametes, the individual level of plasticity in responses, and how elevated temperature may disrupt male–female interactions known to influence fertilization rates in this broadcast spawning invertebrate (Evans et al. 2012; Oliver and Evans 2014). We use a cross‐classified block design to measure how fertilization rates compare between pairs of males and females under contrasting temperatures. This experimental design allows us to partition individual responses to temperature stress and determine the extent of individual plasticity across varying environments. It is important to stress that our design cannot distinguish genetic from environmental effects as we only measure fertilization rates and not offspring traits. Thus, “male” and “female” effects can equally be attributed to genetic impacts or environmental influences, such as age, size, or historical environment. We can, however, determine whether thermal stress affects male–female interactions; a reduction in the ability of gametes to signal or detect compatibility may disrupt mechanisms maintaining heritable variation in fitness traits within populations (Tregenza and Wedell 2000; Puurtinen et al. 2005). Understanding the impacts of rising temperature on fertilization rates is important not only for predicting impacts of climate change on marine broadcast spawners, but could inform practices and management for both marine biofouling and the aquaculture industry (Elliott 2000; Huchette et al. 2004).

## Materials and Methods

### Experimental overview

Fertilizations were undertaken according to a 2 × 2 block cross‐classified mating design, where blocks of two males and two females were crossed in all combinations, with fertilizations each male‐by‐female pair performed in replicate under two temperature conditions (18 and 24°C). This experimental design allows us to partition the underlying variation in fertilization rates among males and females along with their interactions with the environmental treatment. This enables us to determine whether patterns of male‐by‐female interaction at fertilization (indicative of gamete compatibility) are influenced by temperature. We established 10 such blocks of 2 × 2 crosses, arising from 20 males and 20 females.

Winter sea surface temperatures in this region average 18°C (IMOS 2016), but temperatures are predicted to rise up to 3.5°C over the coming century (Hobday and Lough 2011; Lough et al. 2012; IPCC 2013), and nearby heat waves saw sea temperatures rise by 5°C (Pearce and Feng 2013). Adult *Mytilus* spp. show signs of stress when acclimated at temperatures over 25°C (Wallis 1975; His et al. 1989; Anestis et al. 2007; Jansen et al. 2009; Galimany et al. 2011; Sánchez‐Lazo and Martínez‐Pita 2012). Therefore, we have selected our elevated temperature treatment (24°C) to be a realistic future temperature increase that may not greatly impact the more tolerant adults.

### Study species

*Mytilus galloprovincialis* is considered an ecosystem engineer (Gutiérrez et al. 2003), forming large mussel beds that provide habitat for numerous organisms. Mussels were collected by hand from a pontoon at Woodman Point, 30 km south of Perth, Western Australia, from July to September 2014 (permit no. 2141, Department of Transport, Government of Western Australia). Mussels were kept in aerated aquaria of recirculating filtered seawater (FSW) at the University of Western Australia until required (within 1 week of collection).

### Experimental setup

Four water baths – two replicates of each experimental temperature (18°C and 24°C) – were set up in a temperature‐controlled room. In each water bath, four 50‐mL plastic tubes per block were floated in a polystyrene frame. A known volume of 5 mL of FSW was added to each tube and left until the temperature had equilibrated. Water temperature was measured before and after each experimental run using a calibrated pH meter (TPS WP‐81) to ensure it remained constant (Treatment 1 = 17.7°C ± 0.1 SE; Treatment 2 = 23.8 °C ± 0.1 SE).

### Spawning

Mussels were induced to spawn using a temperature shock by moving them from their holding tanks (at ~17°C) to a large tray of shallow FSW preheated to ~24°C using an aquarium heater. We note that while the temperature shock may conceivably influence fertilization rates, this is the standard methodology for spawning *Mytilus*. Spawning mussels were removed from the elevated temperature to jars of ambient FSW to collect gametes, which were later split between experimental treatments. Each mussel was treated alike; thus, any effects of the initial elevated spawning temperature on subsequent fertilization rates would be consistent across treatments, although we concede that any such effect might nevertheless influence our ensuing results.

Male mussels that began spawning were immediately removed from the spawning tray, rinsed in FSW, and then wrapped in wet paper towel to halt spawning until enough eggs had been collected. Each male was then placed in a glass holding jar containing 30 mL of ambient FSW (~17°C) and left to spawn for approximately 10 min. When sperm were sufficiently concentrated (as judged initially by eye), sperm density was estimated using an improved Neubauer hemocytometer (Hirschmann Laborgeräte, Eberstadt, Germany).

Female mussels that commenced spawning were also immediately removed from the spawning tray, rinsed in FSW, then placed in individual glass holding jars each containing 30 mL of ambient FSW (~17°C), and left for approximately 30 min to spawn. When sufficient eggs had been collected, a 5‐*μ*L subsample was counted under a microscope to estimate egg density. Eggs from each female were then split between two plastic tubes per water bath at a density of 5000 eggs per mL – making up the total volume in the tubes to 10 mL – and left for an “incubation phase” of 10 min to ensure water temperatures equilibrated.

### Fertilization

After the 10‐min egg “incubation phase”, an aliquot of sperm from each male was added to the eggs in the plastic tubes according to the crossing design at a density of 50,000 sperm per mL – a ratio previously shown to result in moderate fertilization rates, while avoiding ceiling or basement effects (Oliver and Evans 2014). After 30 min, excess sperm were removed and fertilization halted by rinsing the eggs with temperature‐equilibrated FSW in a 30‐*μ*m mesh sieve. Developing eggs were returned to the experimental tubes and then preserved after 2 h in 1% formalin. The fertilization rate of each replicate was later scored under a microscope as the percentage of eggs showing signs of cleavage and/or with polar body formation among ~100 haphazardly chosen eggs (Longo and Anderson 1969).

### Statistical analyses

Fertilization rate was analyzed as a binomial response trait using a generalized linear mixed‐effects model (GLMM) fit by maximum likelihood (Laplace approximation) with a logit‐link function using the package “lme4” (Bates et al. 2015) in R (R Development Core Team 2016; version 3.2.2). The final model included temperature as a fixed effect, and male and female, as random effects. All interactions involving random effects were included as random factors. An observation‐level random effect was included to deal with overdispersion (Harrison 2014). Wald chi‐square (*Χ* ^*2*^) tests were used to assess the significance of fixed effects, using the R package “car” (Fox et al. 2011), while parametric bootstrapping was used to assess the significance of random effects using the R package “pbkrtest” (Halekoh and Højsgaard 2014). All figures were made using the R package “ggplot2” (Wickham 2009).

## Results

**Figure 1:**
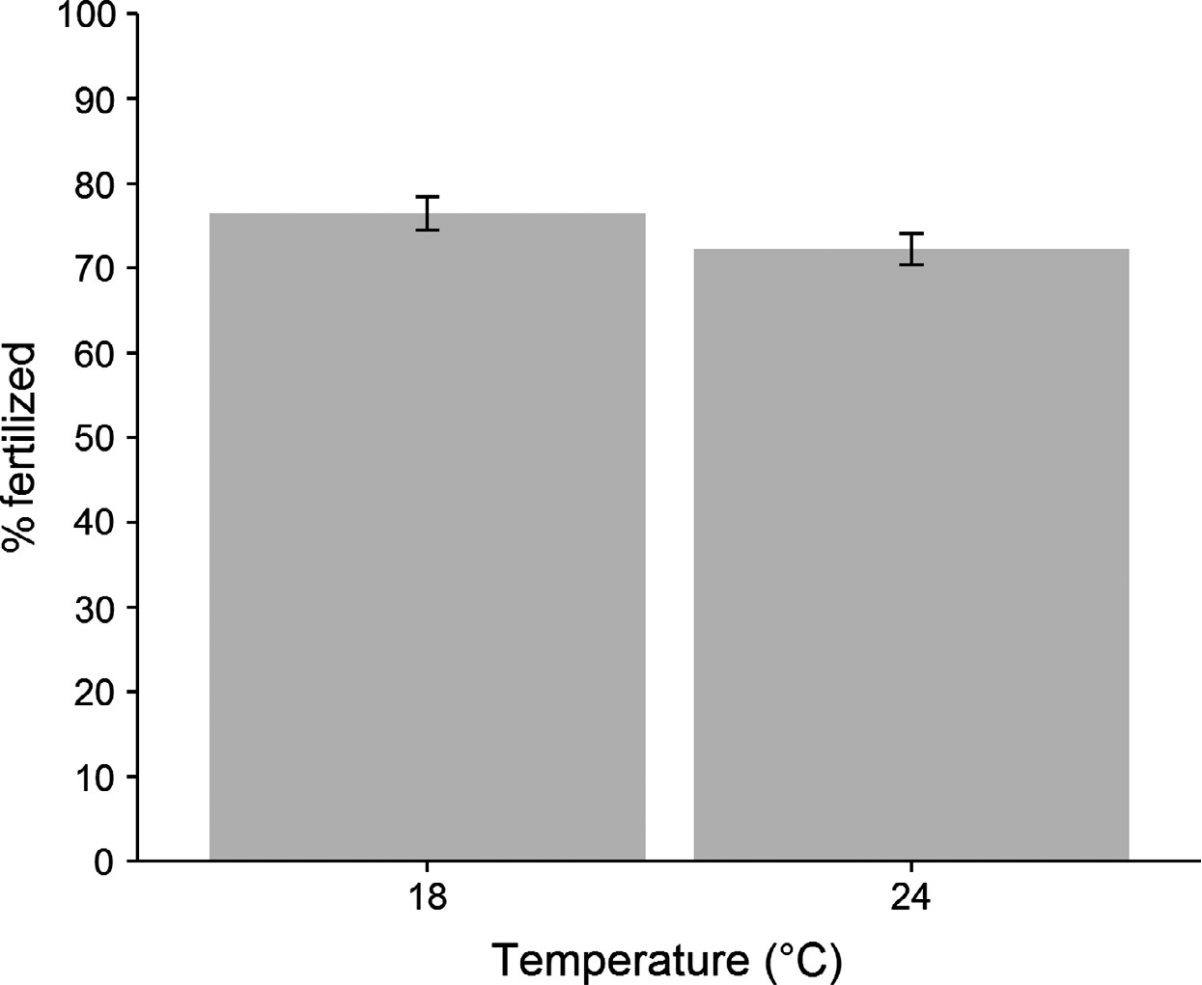
Overall effect of temperature on fertilization rate (±SE).

**Figure 2:**
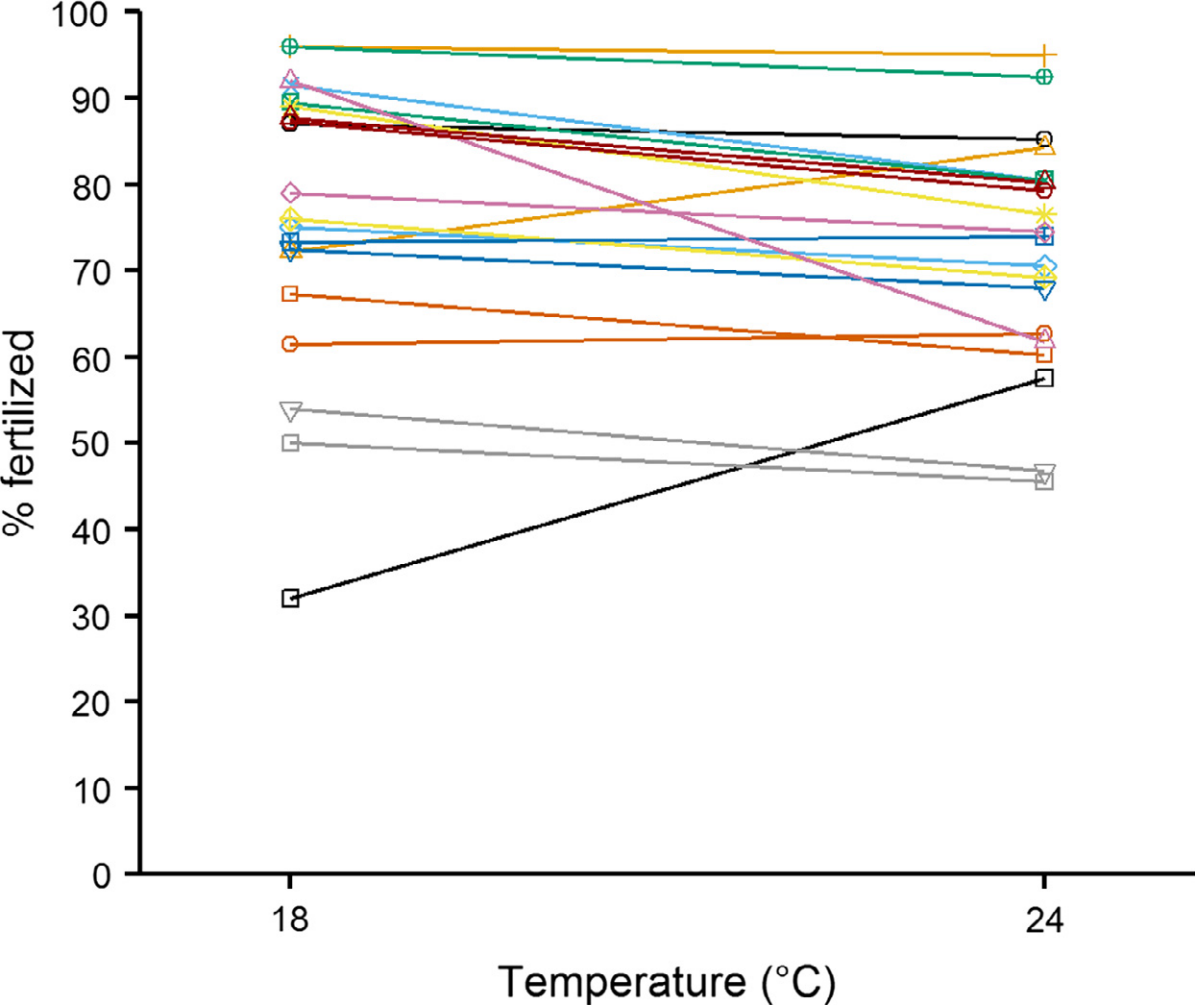
Reaction norm plot showing temperature effect on fertilization rates across individual male mussels (where each line represents a different male; colored by block).

**Figure 3:**
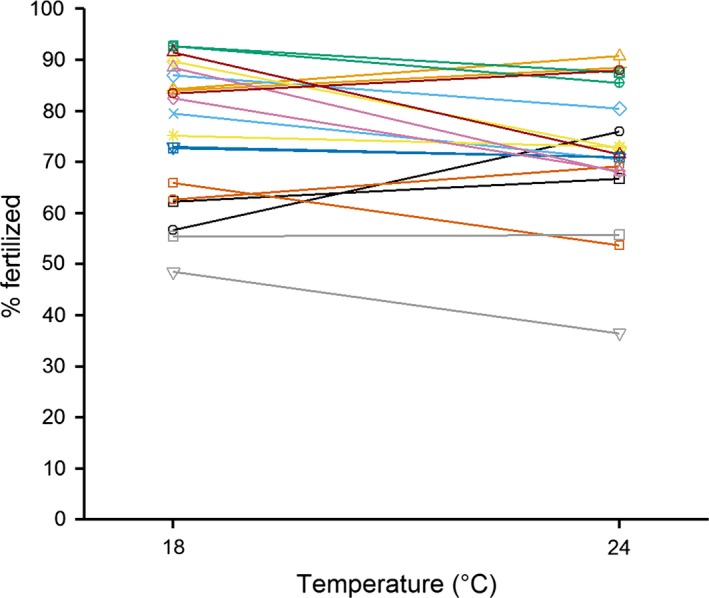
Reaction norm plot showing temperature effect on fertilization rates across individual female mussels (where each line represents a different female; colored by block).

Overall, fertilization rates in the high temperature treatment were significantly lower than those in the low temperature (Fig. 1), but significant male‐by‐temperature (Fig. 2) and female‐by‐temperature (Fig. 3) interactions indicate that the direction of the temperature effect varied among individuals (Table 1). Taken together, this indicates that individual males and females had contrasting fertilizing abilities between the two temperature treatments. Male identity explained significant variation in fertilization rates, while variation attributable to female identity was not significant (Table 1); however, these main effects are difficult to interpret in the presence of significant interactions.

**Table 1:** Temperature, male, and female effects, and their interactions, on fertilization rate as a binomial response in a GLMM

Effect/interaction	Wald Χ^2^/G^2^	*P* value	AIC
Temperature	4.07	*0.04*	16,917
Male	17.87	*<0.01*	16,933
Female	<0.01	0.80	16,915
Male × Female	<0.01	>0.99	16,915
Male × Temperature	6.50	*<0.01*	16,922
Female × Temperature	6.29	*<0.01*	16,921
Male × Female × Temperature	<0.01	0.78	16,915

Significant *P*‐values are italicized.

Surprisingly, we found no strong evidence of a male‐by‐female interaction or three‐way interaction between male, female, and temperature (Table 1). To disentangle whether the large influence of temperature may have swamped more subtle variation due to incompatibility between pairs, we looked at the male‐by‐female interaction within each temperature treatment (Table 2). We found that male and female identity both had a significant influence on fertilization rate within each temperature condition, but there was still no evidence of a male‐by‐female interaction under either condition (Table 2).

**Table 2:** Male and female effects on fertilization rate from a binomial GLMM, modeled within each temperature treatment

Effect/Interaction	18°C		24°C	
	G^2^	*P* value	G^2^	*P* value
Male	31.57	*<0.01*	11.52	*<0.01*
Female	4.87	*<0.01*	3.87	*0.02*
Male × Female	<0.01	0.86	<0.01	0.76

Significant *P*‐values are italicized.

## Discussion

We found that overall fertilization rates were slightly, but significantly, reduced when *M. galloprovincialis* gametes were crossed under temperatures elevated 6°C above ambient. Furthermore, we found that the effect of temperature on fertilization rates varied notably between individuals, with some mussels showing increased fertilization rates with higher temperature, but the majority experiencing the opposite. This result may be indicative of a genotype‐by‐environment interaction (GEI), where particular genotypes have different reaction norms to environmental change. Because *Mytilus* spp. are sessile aquatic organisms with a planktonic larval stage that inhabit highly fluctuating intertidal habitats, we would expect to see large phenotypic plasticity in their responses to environmental changes (Berrigan and Scheiner 2004; Hollander 2008; Gunderson and Stillman 2015). Alternatively, the among‐individual plasticity in fertilization rates may simply be caused by nonheritable variation due to environmental effects, for example, attributable to variation in parent age or size, gamete age, size, or quality, maternal effects, or historical parental environment. In order to discriminate between these two possibilities, a quantitative genetic experiment is necessary (Falconer and Mackay 1996; Lynch and Walsh 1998).

Many studies examining ocean change impacts on marine broadcast spawners use pooled gametes from multiple sires and dams when studying fertilization rates – to reflect the “natural state” of a population broadcast spawning (Byrne 2012) and to avoid incompatible combinations of gametes (Evans and Marshall 2005; Evans et al. 2012). This assesses the average response of a population to ocean changes; however, without considering individual variation in fertilization rates, one may make assumptions for populations that simply reflect successful combinations of the most stress tolerant phenotypes under particular experimental conditions. While our results showed that certain individuals were favored by our experimental treatment, most were susceptible to negative impacts of elevated temperature. These subtle variations are important when considering the capability of marine organisms to adapt to ocean changes, as they may reflect genetic variation for thermal tolerance. Future experimentation should aim to test this directly, as more tolerant mussels could be selectively bred for both aquaculture and conservation purposes.

It has been shown that chemical cues released from *M. galloprovincialis* eggs guide those sperm that result in higher fertilization rates and larval survival toward them (Evans et al. 2012; Oliver and Evans 2014) – an indication that this species can both detect and respond to genetic compatibility signals prior to gamete contact. We anticipated that a change in temperature would result in a breakdown of male‐by‐female interactions, possibly due to the negative impacts of elevated sea temperatures on gamete communication (e.g., differential sperm chemotaxis). However, we found no evidence for male‐by‐female compatibility under elevated or ambient temperatures; thus, we are unable to conclude whether temperature impacted on the previously established compatibility in *M. galloprovincialis* (Evans et al. 2012; Oliver and Evans 2014). As opposed to the “good genes” hypothesis – where a more “fit” male will produce offspring with higher fitness irrespective of the female's genetic quality (Hamilton and Zuk 1982) – the genetic compatibility hypothesis declares that more compatible gametes are attracted to each other to maintain genetic variance in these usually directionally selected fitness traits (Tregenza and Wedell 2000); that is, sperm are not necessarily attracted to eggs that produce offspring that are more “fit” in the traditional sense, but perhaps those that would produce more heterozygous offspring or maintain genetic variation of fitness traits within a population (Mays and Hill 2004; Puurtinen et al. 2005). If this were the case in *Mytilus* spp., it may be that we found no interaction because our experiment was undertaken in small containers and did not allow for competition. This could allow those sperm not detecting or responding to egg cues to be the first to fertilize proximate eggs as “more competitive” sperm were not present. It may also be that the mussels we used comprised no incompatible pairings; certainly, none was completely infertile, although certain individuals consistently produced fertilization rates under 70%. Compatibility effects may also be population dependent, or the population could be inbred due to poor recruitment and unable to detect any differential signaling (Mays and Hill 2004). Patterns of compatibility have been known to differ among populations; for example, in the sea urchin, *Heliocidaris erythrogramma,* a population on the east coast of Australia showed strong compatibility impacts on fertilization rates (Evans and Marshall 2005), while a population on the west coast showed none (Evans et al. 2007).

Two reviews on the effects of ocean climate change on marine invertebrates conclude that fertilization in a range of species is robust to future warming scenarios (Byrne 2011, 2012; but see Byrne and Przeslawski 2013). However, of the included studies that actually reported fertilization rates under experimentally adjusted water temperatures, half found a negative impact of elevated temperatures on fertilization rates (Byrne 2011, 2012). Only two of the six articles on Mollusca included in the 2011 review experimentally altered the temperature conditions under which fertilizations were performed (Clotteau and Dubé 1993; Parker et al. 2010), and the latter found a negative effect of elevated temperature on fertilization rates. We suggest that the fertilization process in broadcast spawners is complex and research in this field needs more subtle distinctions with regard to environmental effects and genetic influences.

Our finding that a relatively tolerant intertidal species such as *M. galloprovincialis* shows decreased fertilization with temperature stress suggests that more sensitive marine invertebrate taxa face severe reproductive costs associated with rising sea temperatures due to climate change. Marine broadcast spawners will be faced not only with elevated temperatures, but also compounding stressors of rising acidity, disease, invasive species, and pollutants (Doney et al. 2012). Even minor impacts to fundamental early lifecycle stages, including fertilization, can lead to major impacts on survival and persistence (Pechenik 2006; Dupont et al. 2010; Byrne 2011; Byrne and Przeslawski 2013). Whether the impacts of elevated temperature on fertilization reported here affect subsequent embryo and larval fitness remains to be investigated. While we report significant plasticity in individual responses to environmental change for one important trait, further exploration is warranted into whether these responses have a genetic basis and thus whether selection can act on such variation.

## Conflict of Interest

None declared.

## Data archiving statement

Data for this study are available at the Dryad Digital Repository (URL supplied after manuscript is accepted for publication).
